# Oxidative Damage and Nrf2 Translocation Induced by Toxicities of Deoxynivalenol on the Placental and Embryo on Gestation Day 12.5 d and 18.5 d

**DOI:** 10.3390/toxins10090370

**Published:** 2018-09-13

**Authors:** Miao Yu, Zhi-Yuan Wei, Zhou-Heng Xu, Jia-Qi Pan, Jian-Huan Chen

**Affiliations:** 1 Genomic and Precision Medicine Laboratory, Department of Public Health, Wuxi School of Medicine, Jiangnan University, 1800 Lihu Road, Wuxi 214122, China; yumiao@jiangnan.edu.cn (M.Y.); 6172805018@stu.jiangnan.edu.cn (Z.-Y.W.); xzh_bio@vip.jiangnan.edu.cn (Z.-H.X.); 6172805016@stu.jiangnan.edu.cn (J.-Q.P.); 2 Guangdong Institute of Applied Biological Resources, 105 Xingang Rd. West, Guangzhou 510260, China

**Keywords:** deoxynivalenol (DON), embryotoxicity, placenta, Nrf2 translocation

## Abstract

Deoxynivalenol (DON) is a kind of natural pollutant belonging to the trichothecenes family. The aim of this study is to use diverse assays to evaluate oxidative damage as well as translocation of nuclear factor erythroid 2-related factor 2 (Nrf2), and to investigate their mechanisms in DON-induced toxicities on a placenta and embryo. Pregnant C57BL/6 mice were randomly assigned to three groups with different doses of DON: 0, 1.0, 2.5 mg/(kg·day). In gestation day (GD) 12.5 d and 18.5 d, DON induced an elevated resorption rate of the embryos as well as structural and functional damage of the placenta. In the placenta, altered levels of the antioxidant enzymes malondialdehyde, superoxide dismutase and glutathione indicated remarkable oxidative stress. Furthermore, an elevated level of heme oxygenase-1 (HO-1) and the translocation of Nrf2 from nucleus to cytoplasm indicated Nrf2/HO-1 pathway activation in DON-L group (1.0 mg/(kg·day)). It is noteworthy that the results in this experiment in GD 12.5 d were similar to those in GD 18.5 d. In conclusion, DON-induced placental oxidative damage and Nrf2 translocation were similar in GD 12.5 d and GD 18.5 d. Oxidative stress is one of the most important molecular mechanisms for embryotoxicity induced by DON, and Nrf2 translocation may play a substantial role against it.

## 1. Introduction

Deoxynivalenol (DON, vomitoxin) is a kind of trichothecenes present widespread in various crops [1], especially in wheat, barley, oats, etc. [2]. DON is a dangerous natural pollutant due to its broad global distribution and extremely high stability [3]. The contamination of DON has also been reported in numerous countries such as the Czech Republic [4], Croatia [5], Canada [6], etc. With its acute toxicities has been widely reported [1,7], its immunotoxicity [2], teratogenicity [8] and carcinogenicity [9] trend becoming visible.

In 1982, the embryotoxicity of DON was reported for the first time [10]. Then studies confirmed that the main toxic effects of DON on a pregnant mother was weight restriction, and on a fetus were abnormal skeletal development [11,12,13], and other disorders that related to pregnancy were reported as well, e.g., abortions, growth restriction [14]. Our previous study showed skeleton abnormalities of the embryos induced by DON [8]. Furthermore, DON-induced oxidative stress could be one of the most critical factors that induce embryotoxicity while this effect could be mediated by Nrf2/HO-1 pathway.

Oxidative stress is an important molecular mechanism of toxicity of DON [1]. Specifically, DON disrupts the mitochondria’s normal function and leads to free radicals (including reactive oxygen species, ROS) accumulation which result in lipid peroxidation and alter the cells’ antioxidant status. Ultimately, these reduce the activities of antioxidant enzymes, including glutathione (GSH) and superoxide dismutase (SOD), etc. [15]. In addition, oxidative stress has been suggested as a causative agent in human and animal pregnancy-related disorders, such as embryonic reabsorption, recurrent pregnancy loss, preeclampsia, intra-uterine growth restriction and fetal death [16]. Heme oxygenase-1 (HO-1) is a heat stress protein which related to anti-inflammatory and antioxidant responses [17]. Various oxidative stimuli (e.g., ROS, inflammatory cytokines and UV irradiation, etc.) can increase the expression of HO-1 [17,18]. HO-1 is the rate-limiting enzyme in the degradation of heme to free iron, carbon monoxide (CO), biliverdin. While the free iron can prevent the aggregation of ROS through a complex set of biochemical processes [19]. It is noteworthy that the expression of HO-1 is regulated by nuclear factor erythroid 2-related factor 2 (Nrf2) binding to its consensus binding sequence [20]. Nrf2, a transcription factor, regulates cellular redox balance and protects phase II detoxification and antioxidant responses [21]. Under quiescent conditions, a dimer of Nrf2 and Keap1 exists in the cytoplasm. Once exposure to electrophiles or ROS, Nrf2 dissociates from Keap1 and translocates from cytoplasm into the nucleus, and activates transcription of various cytoprotective genes including HO-1 [22]. In a recent study, DON has been confirmed monitoring the nuclear translocation of Nrf2 in the human Jurkat T-cell line [23]. The study was an in vitro experiment, our study would focus on effects of DON on Nrf2 activity in vivo.

Although our previous study explored the above mechanism for the first time, further studies are needed to further elucidate it. In the current study, we used the mouse model that used in our previous study [8] to show the embryonic developmental toxicities of DON, and focused on the comparison of DON’s toxicities on gestation day (GD) 12.5 d and 18.5 d both in the placenta and embryo. GD 9.5–11.5 d is the critical period of development during pregnant, and thus is chosen as the exposure time. Examination of the placenta and embryo in GD 12.5 d can be used to assess the acute response of the mother and fetus to DON, and is compared to that in GD 18.5 d.

Therefore, we examined the placenta and embryo on GD 12.5 d and 18.5 d. Histopathological observation and the level of β-chorionic gonadotrophin (β-CG) were used to evaluate the placental structure and function situation respectively. The rates of resorbed, dead and live embryos were the reflection of embryonic survival, growth and development. The oxidant and antioxidant level of placenta was expressed using ROS, malondialdehyde (MDA), SOD, GSH. The level of HO-1 and translocation of Nrf2 showed the expression of Nrf2/HO-1 signal pathway. The current study the first time reveals the changes in placenta after DON exposure from GD 12.5 d to 18.5 d. These results present basic data for risk assessment of embryotoxicity induced by DON.

## 2. Results

### 2.1. Effect of DON Exposure on Body Weight

**Figure 1:**
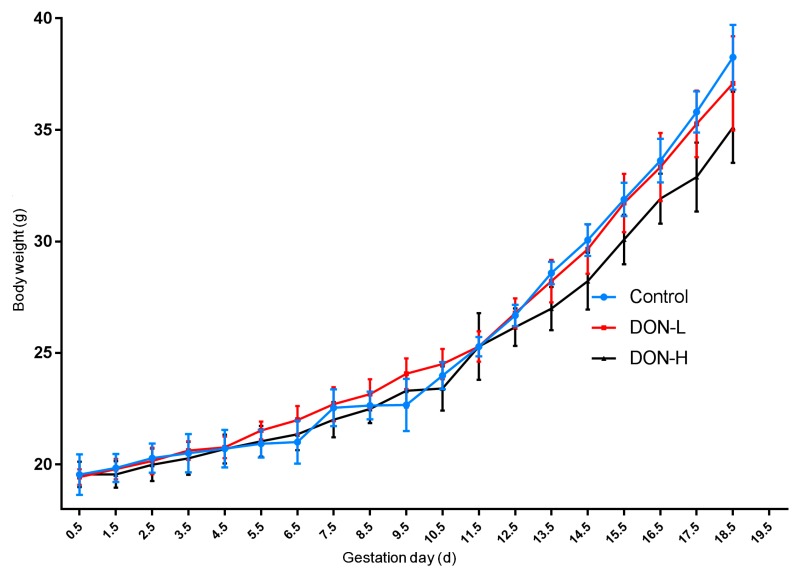
Mean body weight of pregnant mice in different groups. Values were expressed as means ± SEM (*n* = 6). SEM: Standard Error of Mean.

All mice survived until the final sacrifice (GD 12.5 d and GD 18.5 d), and no clinical signs were observed through the study. Body weight was shown in Figure 1, and no significantly difference among all groups was found throughout the whole study. Although the weight increase in DON-H group was relatively slower than the other two groups, this change was not significant.

### 2.2. Effect of DON Exposure on Embryonic Survival, Growth, and Development on GD 12.5 d and GD 18.5 d Embryos

The results of resorbed embryos, dead embryos and live embryos were shown in Table 1. The resorption rates in the DON-treated group (except DON-L in GD 12.5 d) were significantly higher than in the control, while no significant differences were observed when compared the dead rate among all DON-treated groups with the control.

### 2.3. Effect of DON Exposure on Placenta Histopathological and Function Changes in GD 12.5 d and GD 18.5 d Maternal Mice

**Figure 2:**
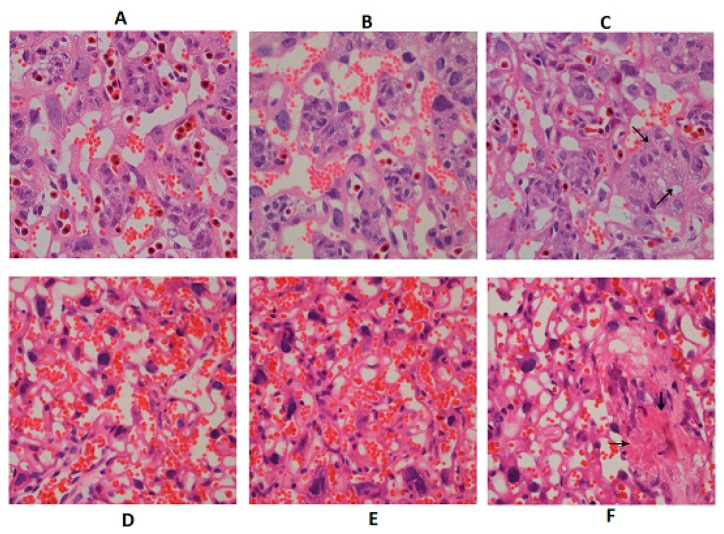
Histopathological changes of placenta of the maternal mice on gestation day (GD) 12.5 d and 18.5 d. GD 12.5 d: (**A**) Control, (**B**) DON low-dose group (DON-L), (**C**) DON high-dose group (DON-H); GD 18.5 d: (**D**) Control, (**E**) DON-L, (**F**) DON-H. Stained with hematoxylin and eosin (H&E, 400×).

Hematoxylin and eosin (H&E) results were shown in Figure 2. Compared to the GD 12.5 d placenta (Figure 2A–C), larger number of blood sinus and more blood flow were found in GD 18.5 d placenta (Figure 2D–F). The placental morphology in the control and DON-L groups appeared to be normal both in GD 12.5 d and GD 18.5 d. However, for the DON-H groups in both GD 12.5 d and GD 18.5 d, decreased number of blood sinus and blood flow were found, and inflammatory cell infiltration was observed (see arrows in Figure 2C,F). In addition, vacuole-like changes, apoptosis and necrosis could be observed in some placenta syncytiotrophoblast.

**Figure 3:**
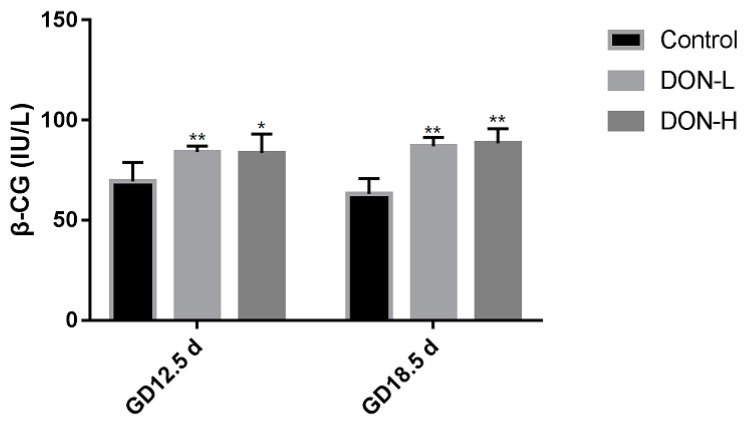
The level of β-chorionic gonadotropin (β-CG) in the placenta on GD 12.5 d and GD 18.5 d. Values were expressed as means ± SEM (*n* = 6). Significant statistical difference compared to the control: * *p* < 0.05 and ** *p* < 0.01.

In addition to blood supply, the level of β-CG is another biomarker to reflect placental function. The level of β-CG was shown in Figure 3 on GD 12.5 d and GD 18.5 d, the level of β-CG in DON-treated groups were significantly higher than in the control (*p* < 0.05 in DON-H group on GD 12.5 d, and others all *p* < 0.01).

### 2.4. Effect of DON Exposure on the Levels of Oxidant and Antioxidant Biomarkers in the Placenta of GD 12.5 d and GD 18.5 d Maternal Mice

**Figure 4:**
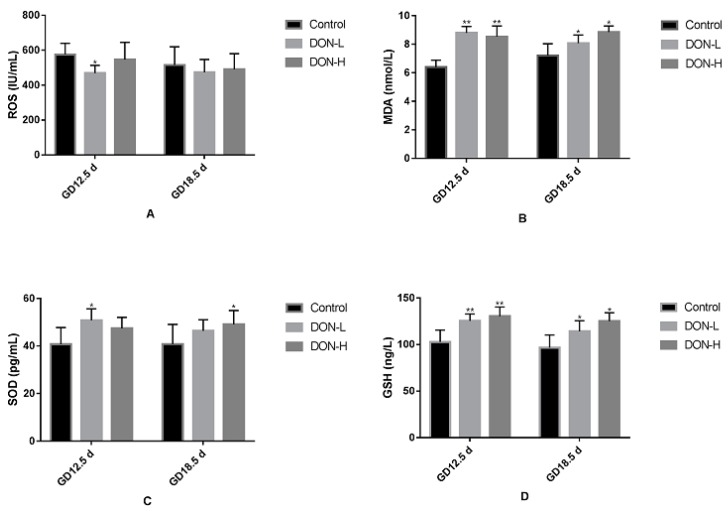
Activities of anti-oxidative system in the placenta on GD 12.5 d and GD 18.5 d. (**A**) ROS; (**B**) MDA; (**C**) SOD; (**D**) GSH. Values were expressed as means ± SEM (*n* = 6). Significant statistical difference compared to the control: * *p* < 0.05 and ** *p* < 0.01.

The levels of ROS, MDA, SOD and GSH were shown in Figure 4. On GD 12.5 d and GD 18.5 d, the levels of ROS in DON-treated groups were lower than in the control, however, it was significant only in DON-L group on GD 12.5 d (*p* < 0.01). MDA and GSH in all DON-treated groups were significantly higher than in the control, on GD 12.5 d and on GD 18.5 d (*p* < 0.01 and *p* < 0.05 respectively). SOD in all DON-treated groups were higher than in the control, but it was significant only in DON-L group on GD 12.5 d and in DON-H group on GD 18.5 d (both *p* < 0.05).

### 2.5. Effect of DON Exposure on the HO-1 Level in Maternal Placenta on GD 12.5 d and GD 18.5 d

**Figure 5:**
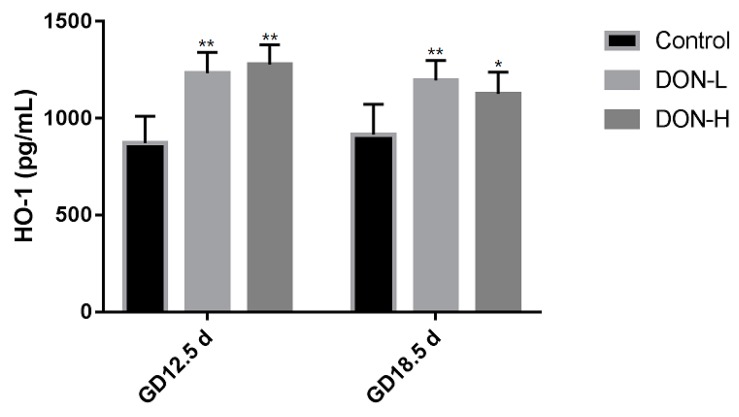
The level of HO-1 in the placenta on GD 12.5 d and GD 18.5 d. Values were expressed as means ± SEM (*n* = 6). Significant statistical difference compared to the control: * *p* < 0.05 and ** *p* < 0.01.

The level of HO-1 was shown in Figure 5. HO-1 in all DON-treated groups were significantly higher than in the control (*p* < 0.05 in DON-H group on GD 18.5 d, *p* < 0.01 in all of the other DON-treated groups).

### 2.6. Effect of DON Exposure on the Intracellular Translocation of Nrf2 in the Placenta of GD 12.5 d and GD 18.5 d Maternal Mice

**Figure 6:**
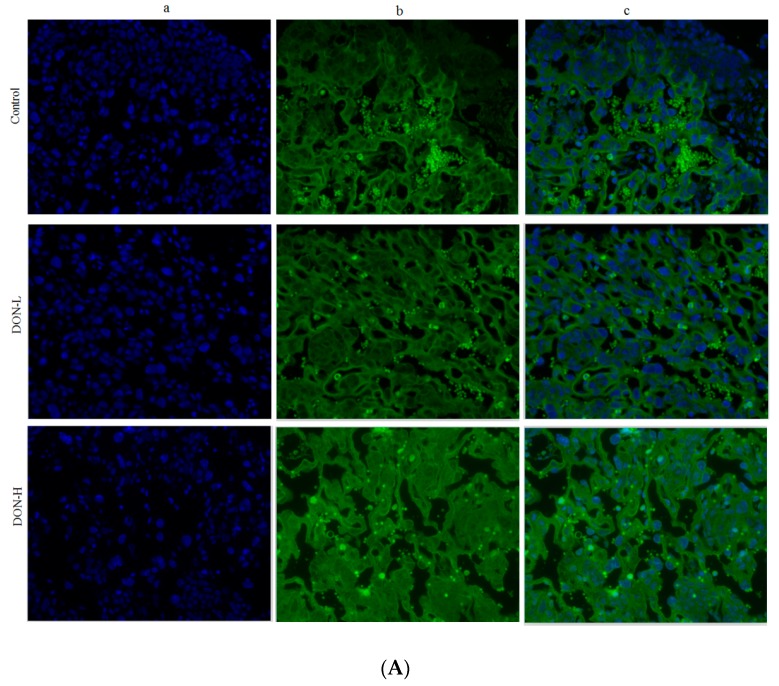

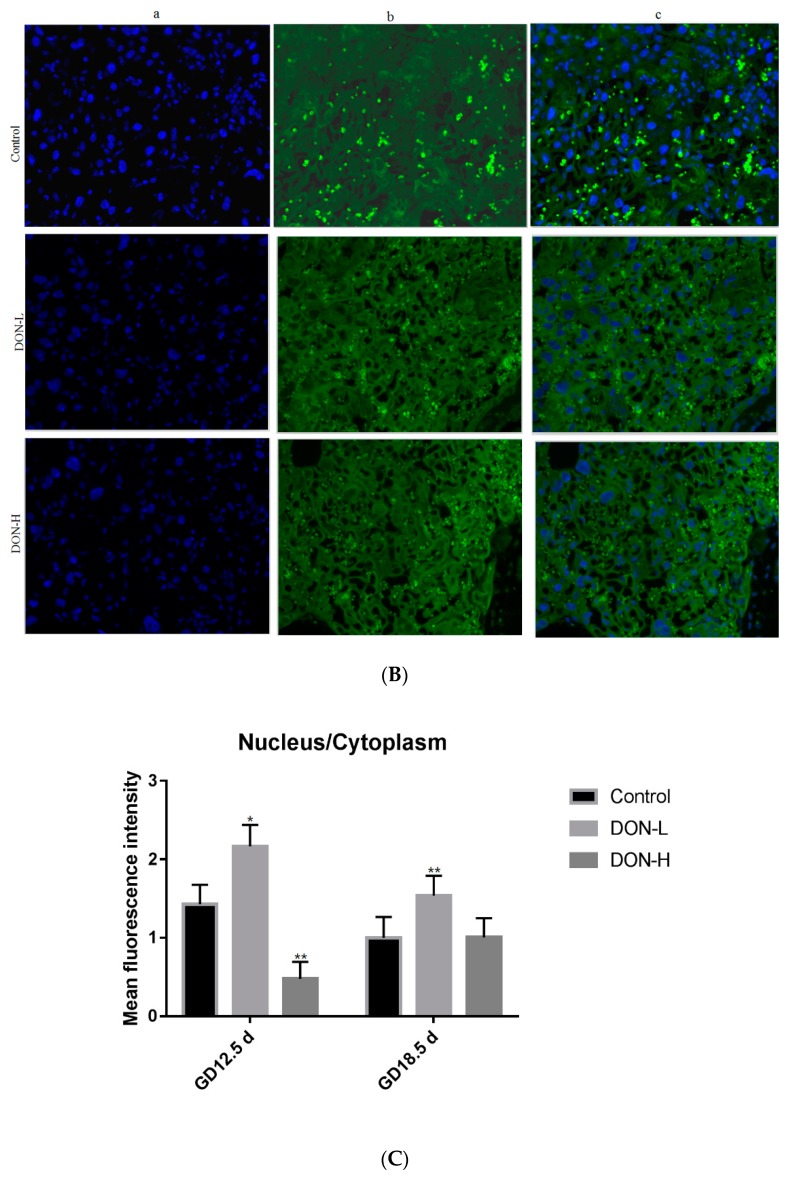
Immunofluorescent findings of Nrf2. Nrf2 in the nucleus (a, 400×); and cytoplasm (b) in the placenta on GD 18.5 (400×); and (c, 400×) an overlay of a and b on GD 12.5 d (**A**) and on GD 18.5 d (**B**). Mean fluorescence intensity (MFI) were assayed using Image-pro Plus 6.0 (Media Cybernetics, Inc. Rockville, MD, USA). The ratio of nucleus/cytoplasm were expressed as mean ± SEM (*n* = 4) (**C**). Significant statistical difference compared to the control: * *p* < 0.05 and ** *p* < 0.01.

The Nrf2 translocation was shown in Figure 6. Mean fluorescence intensity (MFI) in the DON-L group was significantly higher than in the control on both GD 12.5 d (*p* < 0.05) and GD 18.5 d (*p* < 0.01), while MFI in DON-H group on GD 12.5 d was markedly lower than in the control (*p* < 0.01).

## 3. Discussion

Embryotoxicity of DON has received increasing attention in recent studies. In the current study, we revisited the model of DON’s embryotoxicity to mouse and focused on the observation of placenta and embryo on both GD 12.5 d and GD 18.5 d. The effect of DON on embryonic survival, growth and development has been proven in the previous study [10], and our result of resorbed embryos rate was in line with the previous literature. Compared with the previous study, our experiment also examined the resorbed embryos rate on GD 12.5 d. Our result first demonstrates that embryos were resorbed early on GD 12.5 d when exposed pregnant mice in DON during GD 9.5–11.5 d. Furthermore, the rate of resorbed embryos was significant not only in the DON-H group, but also in the DON-L group, suggesting that a low dose of DON (1.0 mg/(kg·day)) might be enough to induce embryos to resorb. Another embryotoxicity of DON is to induce skeleton abnormalities which has been proven in our previous experiment [8]. However, as the skeleton of a normal embryo is not fully developed by GD 12.5 d, this index is not included in the current study.

Apart from the toxicities of DON on embryo, the other aim of the study is to concern the toxicities of DON on the placenta. Our results shows clear structural damage in the placenta, and another index β-CG was also evaluated. The human chorionic gonadotropin (β-HCG) is produced by placental syncytiotrophoblasts, and its role could be mainly autoregulatory and exerting growth-promoting activity [24]. It has been demonstrated that elevated β-HCG may reflect early placental damage or dysfunction [25,26] and villous syncytiotrophoblast in the placenta is the main site for synthesis of HCG [27,28]. Therefore, we measured the level of β-CG in mouse placenta to evaluate placental function. Our findings showed elevated β-CG in the placenta induced by DON exposure both on GD 12.5 d and GD 18.5 d, which indicated placental damage or dysfunction. In conclusion, DON exposure of pregnant mice resulted in resorbed embryos, skeleton deformities of embryos, and structural and functional damage of the placenta.

Our hypothesis of the molecular mechanisms of DON’s embryotoxicity is that DON exposure may lead to ROS accumulation in the placenta, which result in placental oxidative damage and related deficiency of embryo development. To confirm this hypothesis, we firstly measured oxidant and antioxidant markers. ROS is critical for the regulation of embryonic development because of its function to maintain the stability of internal redox environment [14]. Many studies have reported that placenta ROS excessive accumulation induces numerous adverse outcomes of pregnancy [29,30]. Moreover, MDA was one of the most often analyzed markers of oxidative stress over the years. In our current study, MDA in all DON-treated groups were significantly higher while the level of ROS in DON-treated groups were lower than in the control. Meanwhile, the levels of antioxidant indexes SOD and GSH significantly increased. A possible explanation to this is that, DON exposure in pregnant mice probably induced ROS generation in placenta. Then antioxidant system was activated, with the levels of SOD and GSH increased, and eventually led to decrease of ROS. The products of lipid peroxidation, MDA, could be a plausible indicator of ROS-dependent tissue damage [31]. Elevated MDA in DON-treated groups indicated ROS-dependent placental tissue damage. With all of these changes together, it’s surprising that the results in GD 12.5 d were similar with them in the GD 18.5 d. We speculate that the oxidative damage of placenta appears right after the DON exposure and continued until the end of pregnancy. However, further experiment will be needed to verify this speculation.

Oxidative stress, an imbalance between pro-oxidative and anti-oxidative forces in biological systems, is considered to be responsible for the initiation or development of pathological processes affecting female reproductive processes [32]. Nrf2/HO-1 pathway is one of the most critical pathways which can protect cells from oxidative damage to some extent. Therefore, in recent years, the relationship between Nrf2/HO-1 pathway expression and the pregnant outcomes has drawn increasing attention. A study revealed that cigarette smoke might inhibit the development of placenta via activation of ROS by inducing apoptosis and autophagy by affecting the expression of Keap1-Nrf2 expression [33]. In women with both type 2 diabetes mellitus and obesity, oxidative stress detrimentally alters placenta function and increase metabolic disturbance susceptibility in their offspring, in which the Nrf2/HO-1 pathway is involved [34]. Moreover, maternal fructose intake provoked an imbalanced redox status in placenta and a clear diminution of HO-1 expression, which could be responsible for the augmented oxidative stress found in their fetuses [35]. As a result, some antioxidants have been applied to upregulate the expression of Nrf2/HO-1 pathway to protect placenta from oxidative damage. Dietary resveratrol, an antioxidant, was found regulating placental antioxidant genes expression by the Keap1-Nrf2-HO-1 pathway in placenta and features of placental and endothelial dysfunction characteristic of preeclampsia were improved [36,37].

Although the relevance of Nrf2/HO-1 pathway and the pregnant outcomes has been widely discussed, direct evidence of Nrf2 translocation and the pregnant outcomes is limited. A study in vitro found that melatonin improved endothelial function and prolonged pregnancy in women with early-onset preeclampsia through reduced oxidative stress and enhanced antioxidant markers (Nrf2 translocation, HO-1) [38]. In trophoblast cells, it is demonstrated that chlorpyrifos alters the redox balance by inducing an adaptive activation of the Nrf2 translocation with an increase in HO-1 gene. And the adaptive activation of the Nrf2/ARE pathway could confer fetal protection against xenobiotics injury in placenta [39]. However, experiments (in vivo) are needed to connect Nrf2 translocation with the placenta function (pregnant outcomes). In our study, the increased HO-1 level in placenta in all DON-treated groups demonstrated HO-1 might play an important role. In addition, we have implicated the Nrf2/ARE pathway expression in the embryotoxicity of DON in our previous study [8]. Observed translocation of Nrf2 in the present study confirmed the previous findings that DON-induced ROS accumulation led to oxidative stress in placenta. However, in DON-L group, ROS activated the Nrf2 translocation, and the upregulation of HO-1 protected placenta against oxidative damage. However, the frequency of Nrf2 translocation in longer time and higher dose were lower than the other groups. The reason might be that at the shorter time exposure and with the DON-L doses, the cells are in a compensatory state, so the obvious translocation of Nrf2 can be observed. Nevertheless, as the exposure time and dose increased and exceeded the threshold for normal Nrf2 function, the structure and function of cells are impaired, the translocation of Nrf2 disappeared. Our findings thus suggested that applying antioxidants to upregulate the expression of Nrf2/HO-1 pathway could be one of the most useful methods to protect placenta from DON-induced oxidative damage during pregnancy. Our results warrant future study on the Nrf2/HO-1 pathway in DON-induced oxidative damage during pregnancy.

## 4. Materials and Methods

### 4.1. Chemicals

DON (12,13-epoxy-3,4,15-trihydroxytrichotec-9-en-8-one, C_15_H_20_O_6_, MW: 296.32, CAS RN: 51481-10-8, purity ≥99%) was purchased from Sigma-Aldrich (St. Louis, MO, USA). The melting point is 151–153 °C. The ELISA kits of β-CG, HO-1, ROS, MDA, SOD, GSH were acquired from the Huyu Biological Technology Co., Ltd. (Shanghai, China).

### 4.2. Animals and Treatment

C57BL/6 mice (adult female: 64; adult male: 32) were procured from Lingchang BioTech Co., Ltd. (Suzhou, China) and used after one week acclimatization. A specific pathogen-free (SPF) animal facility with strictly controlled environment was used to feed the animals. The feed and tap water were provided ad libitum, whereas the chip bedding were replaced twice a week. The study strictly followed the Guide for the Care and Use of Laboratory Animals, National Institute of Health, 1996, Bethesda, MD, USA. Animal experiments were approved by the Institutional Animal Care and Use Committee at Jiangnan University (Ethical approval code: JN.NO20180115c1440619, Date of approval: 12 January 2018).

### 4.3. Study Design

Eight-week-old female mice were mated with males (female:male = 2:1). Once a vaginal plug was detected in a female mouse, then gestation day 0.5 (GD 0.5 d) was designated. The pregnant mice were randomly divided into three groups according to our previous study [8]: high-dose group (2.5 mg/(kg·day), DON-H), low-dose group (1.0 mg/(kg·day), DON-L) and control group (0 mg/(kg·day), control). We deleted the 5.0 mg/(kg·day) group due to its extremely high absorbed rate (73.1%). DON (dissolved in ultrapure water) was given to the pregnant mice at GD 9.5–11.5 d by gavage. Half of them in each group were sacrificed by cardiac perfusion at GD 12.5 d, and the rest were sacrificed at GD 18.5 d. Rapidly separated the placenta and stained 3 per liter with hematoxylin and eosin (H&E). The rest were quick-frozen with liquid nitrogen for 15 min and then stored at −80 °C until analysis.

### 4.4. β-CG, ROS, MDA, SOD, GSH, and HO-1 in the Placenta

Firstly, placenta tissue was homogenized in ice-cold 50 mM phosphate buffer (pH 7.0) using a Teflon pestle connected to a Braun homogenizer motor (Frankfurt, Germany). The homogenate was then centrifuged at 3500× *g* (Eppendorf centrifuge 5804R, Hamburg, Germany, 4 °C, 10 min) in order to remove all the cell debris and nuclei. Finally, the final supernatant was stored at −20 °C for the following biochemical assays. The levels of β-CG, ROS, MDA, SOD, GSH and HO-1 were measured using commercial Elisa kits (Huyu Biological Technology Co., Ltd., Shanghai, China). The measurements were conducted according to the manufacturer’s specifications. The results of β-CG, ROS, MDA, SOD, GSH and HO-1 are expressed as IU/L, IU/mL, nmol/L, pg/mL, ng/L and pg/mL, respectively.

### 4.5. Placental Pathological Examination and Immunostaining

Placenta tissue was fixed in 10% formalin (24 h), embedded in paraffin, and sectioned at 4 µm. Then, the lacenta sections were deparaffinized, rehydrated, and stained with H&E. Standard immunohistochemical and immunofluorescent staining were performed as described previously [40]. The placental sections were incubated with rabbit monoclonal Nrf2 antibody (1:100, Abcam, Cambridge, MA, USA) overnight at 4 °C. Then, secondary antibodies, goat anti-rabbit antibody (1:200, Abcam, Cambridge, MA, USA) were applied and counterstained with 4,6-diamidino-2-phenylindole (DAPI, 0.0002% solution, Sigma-Aldrich). Images were acquired using an Axio Imager Z2 microscope (Carl Zeiss AG, Heidenheim, Germany). And the expression of Nrf2 was calculated with the Image-Pro Plus 6.0 (Media Cybernetics, Inc. Rockville, MD, USA).

### 4.6. Statistical Analysis

Statistical procedures were carried out with the SPSS 12.0 software for Microsoft Windows (SPSS Inc., Chicago, IL, USA). The results were expressed as means ± SEM. *p* value < 0.05 was considered statistically significant. For comparison between groups, ANOVA was used. Once a statistically significant difference was found, Dunnett’s multiple tests would be used to compare the differences between control and the other groups.

## 5. Conclusions

DON-induced placenta oxidative damage and Nrf2 translocation were similar in GD 12.5 d and GD 18.5 d. Oxidative stress is one of the most important molecular mechanisms for embryotoxicity induced by DON, and Nrf2 translocation may play a substantial role against it.

## Figures and Tables

**Table 1:** Effects of DON on GD 12.5 d and GD 18.5 d embryos.

Group	*N*	Resorbed Embryos (%)	Dead Embryos (%)	Live Embryos (%)
**E12.5 d**				
Control	6	2.0 (1/51)	0 (0/51)	98.0 (50/51)
DON-L	6	17.6 ** (9/51)	2.0 (1/51)	80.4 (41/51)
DON-H	6	28.0 ** (14/50)	6.0 (3/50)	66.0 (33/50)
**E18.5 d**				
Control	6	2.1 (1/48)	0 (0/48)	97.9 (47/48)
DON-L	6	10.2 (5/49)	0 (0/49)	89.8 (44/49)
DON-H	6	18.8 ** (9/48)	2.1 (1/48)	79.1 (38/48)

Values were expressed as means ± SEM, significant statistical difference: ** *p* < 0.01 versus the Control. *N*: number of pregnant mice that examined.

## Abbreviations

β-CG	β-chorionic gonadotropin
β-HCG	β-human chorionic gonadotropin
DON	Deoxynivalenol
DON-H	DON high-dose group
DON-L	DON low-dose group
GD	Gestation day
GSH	Glutathione
H&E	Hematoxylin and eosin
HO-1	Heme oxygenase-1
MDA	Malondialdehyde
MFI	Mean fluorescence intensity
Nrf2	NF-E2-related factor 2
ROS	Reactive oxygen species
SPF	Specific Pathogen Free
SOD	Superoxide Dismutase
